# Supplementary material for: SMARCD3 Promotes Epithelial–Mesenchymal Transition in Gastric Cancer by Integrating PI3K-AKT and WNT/β-Catenin Pathways
Source: Cancers (Basel). 2025 Oct 31;17(21):3526. doi: 10.3390/cancers17213526 (PMC12607331; doi:10.3390/cancers17213526)
Supplement: Supplementary file 1 [file cancers-17-03526-s001.zip › cancers-3939929-supplementary.pdf]

supplementary materials

Western Blot Original Figures

We used protein size marker GenDEPOT(P8502)

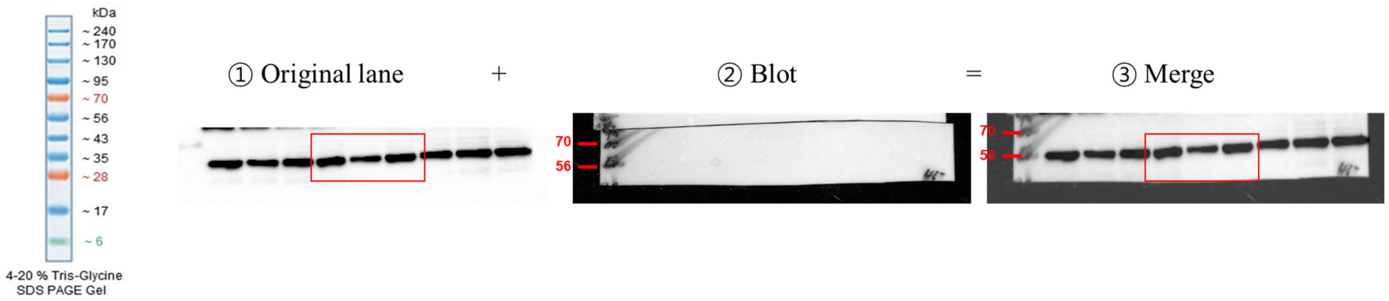

Figure S1. Layout of Western blot and protein marker ①Original lane + ② Blot = ③ Merged figure the following ② Blot are ommitted.

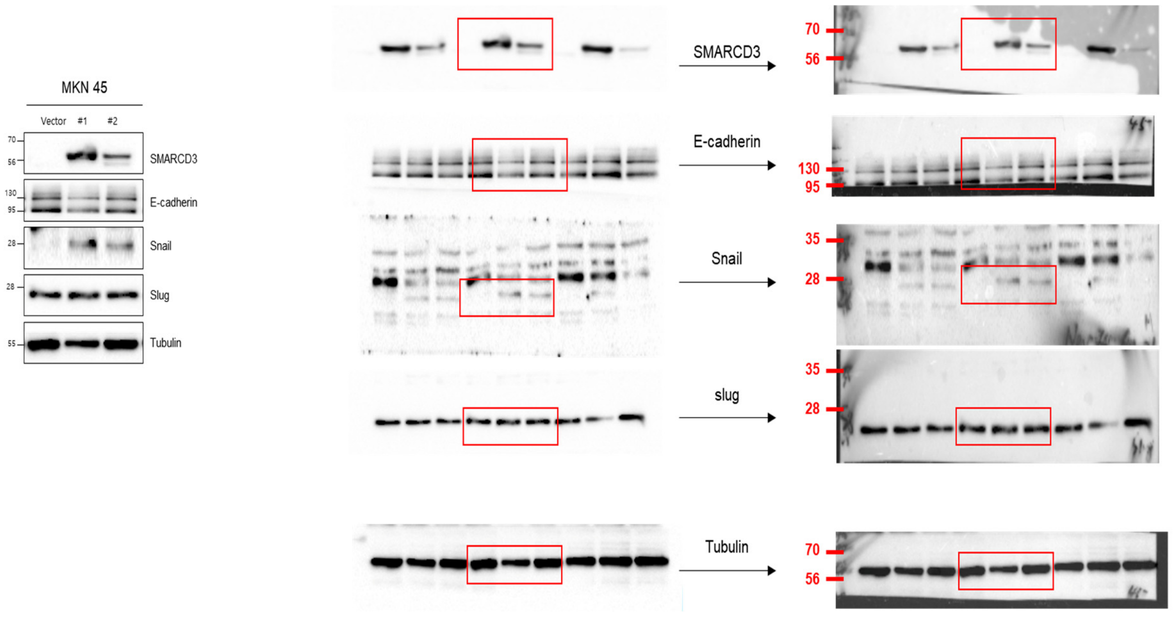

Figure S2. Original Western Blot Figures of Figure 2A.

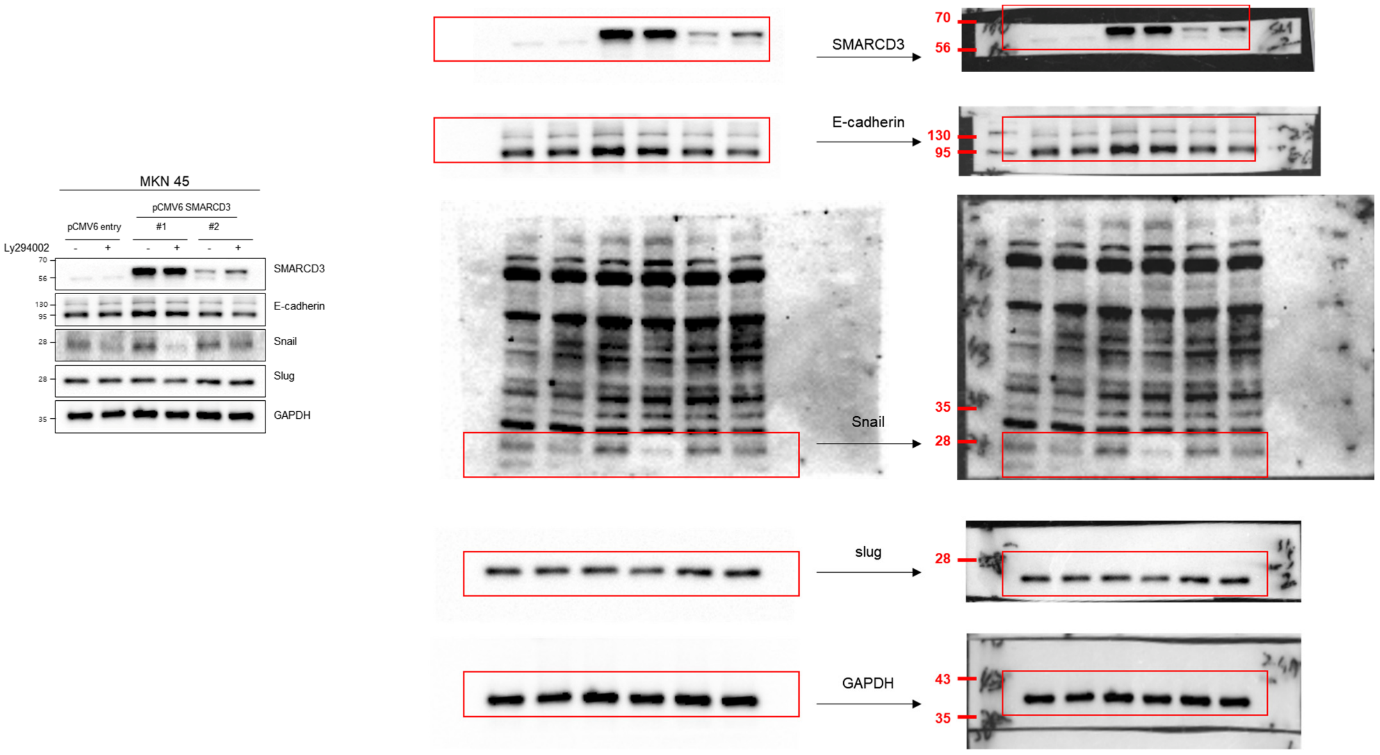

Figure S3. Original Western Blot Figures of Figure 2B.

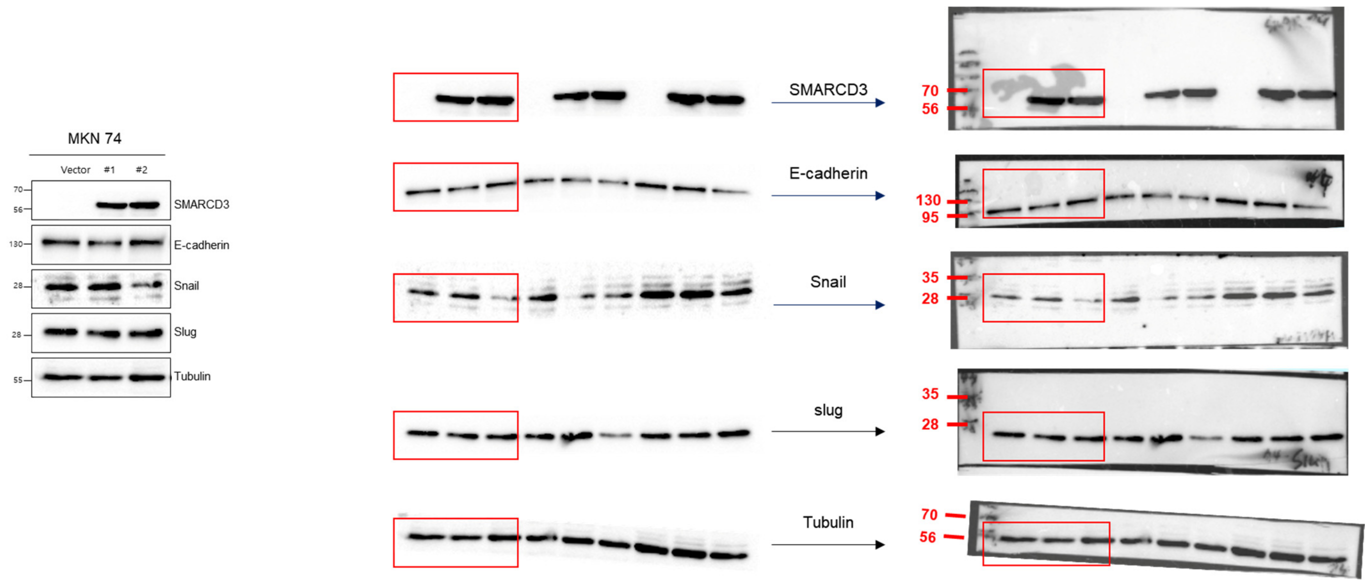

Figure S4. Original Western Blot Figures of Figure 2C.

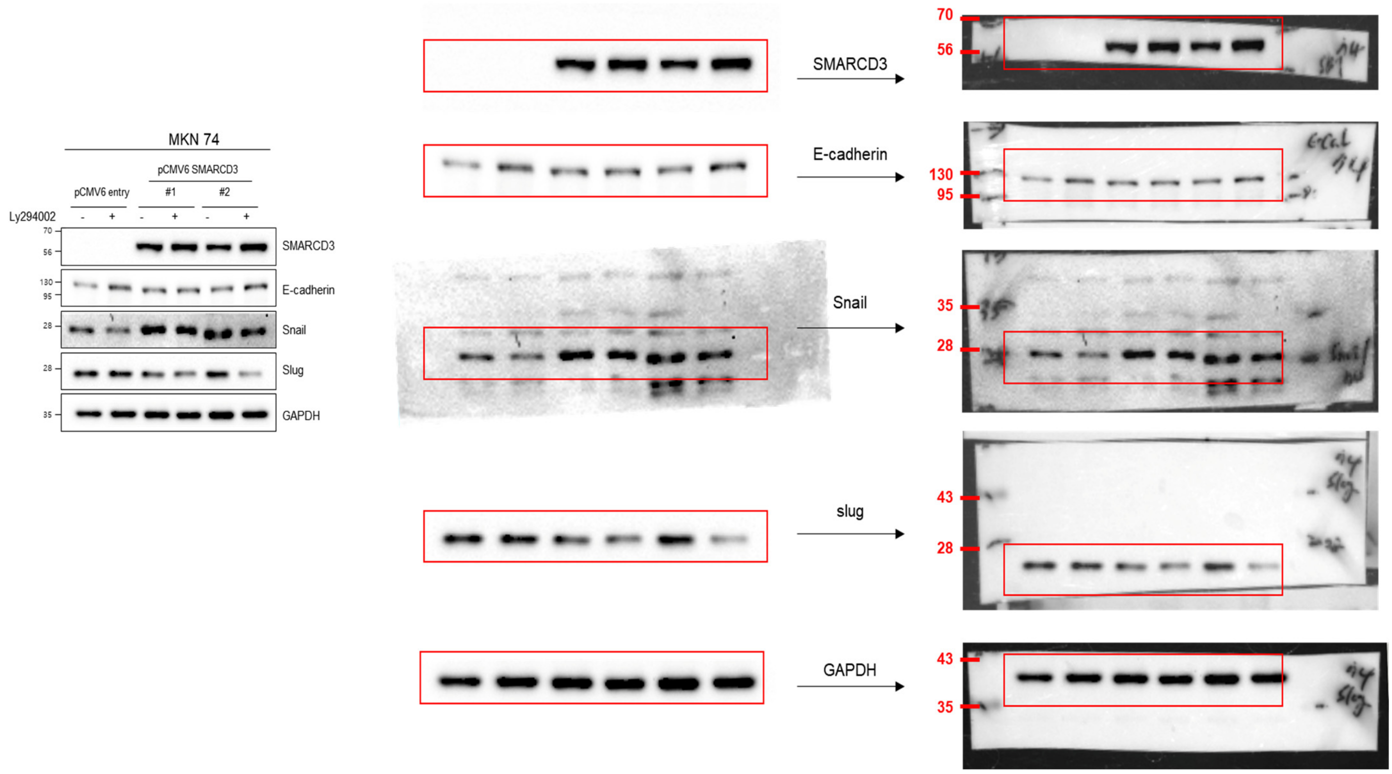

Figure S5. Original Western Blot Figures of Figure 2D.

A

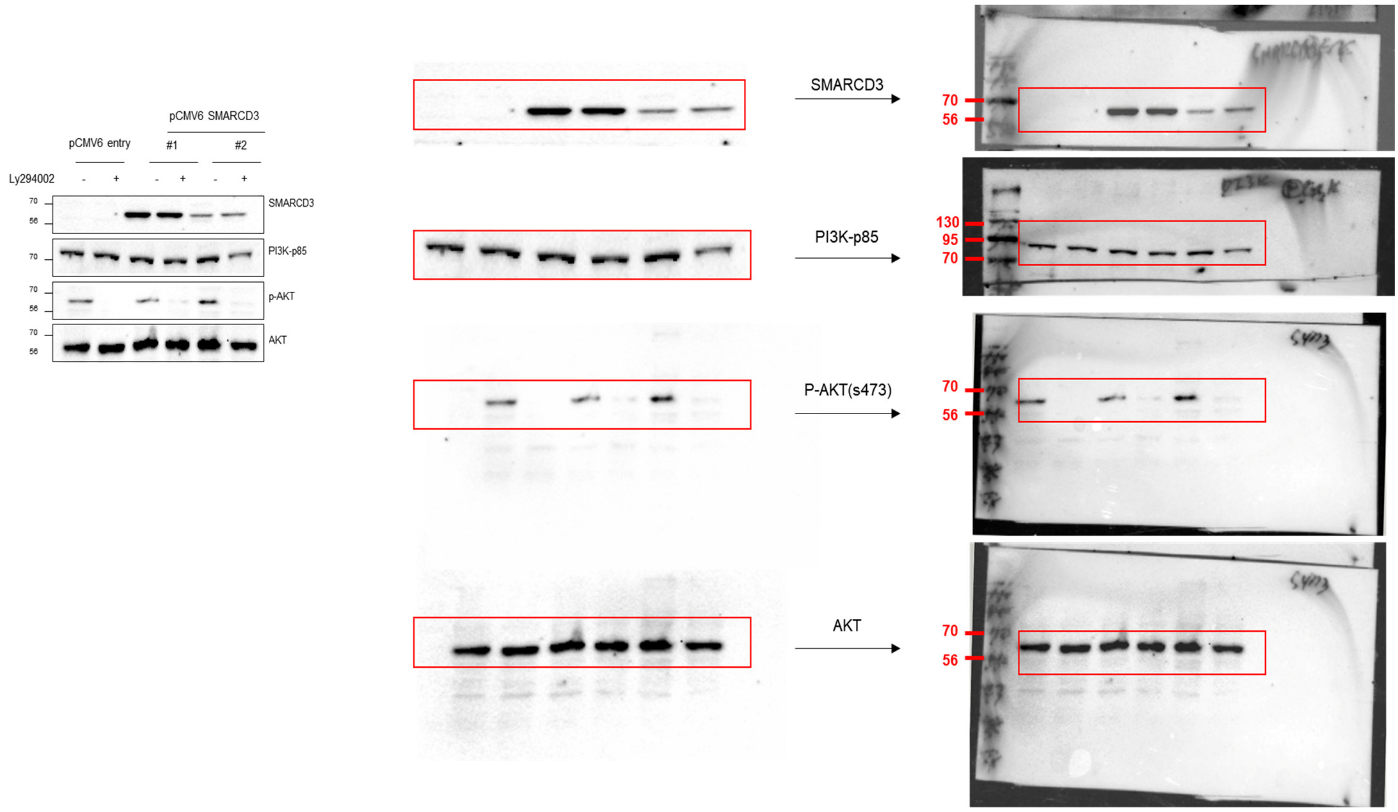

B

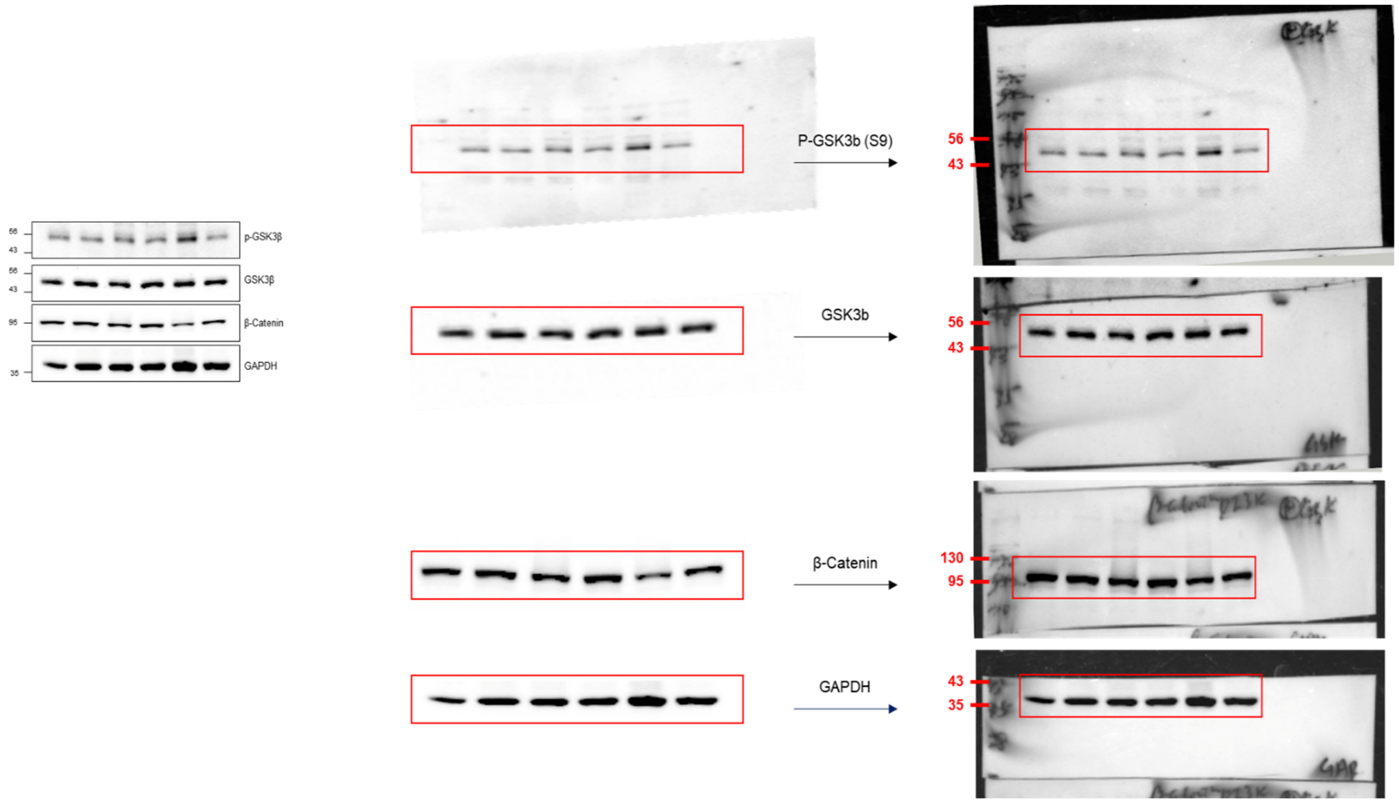
56
43
p-GSK3β
GSK3β
95
β-Catenin
35
GAPDH
P-GSK3b (S9)
GSK3b
β-Catenin
GAPDH
56
43
130
95
35

C

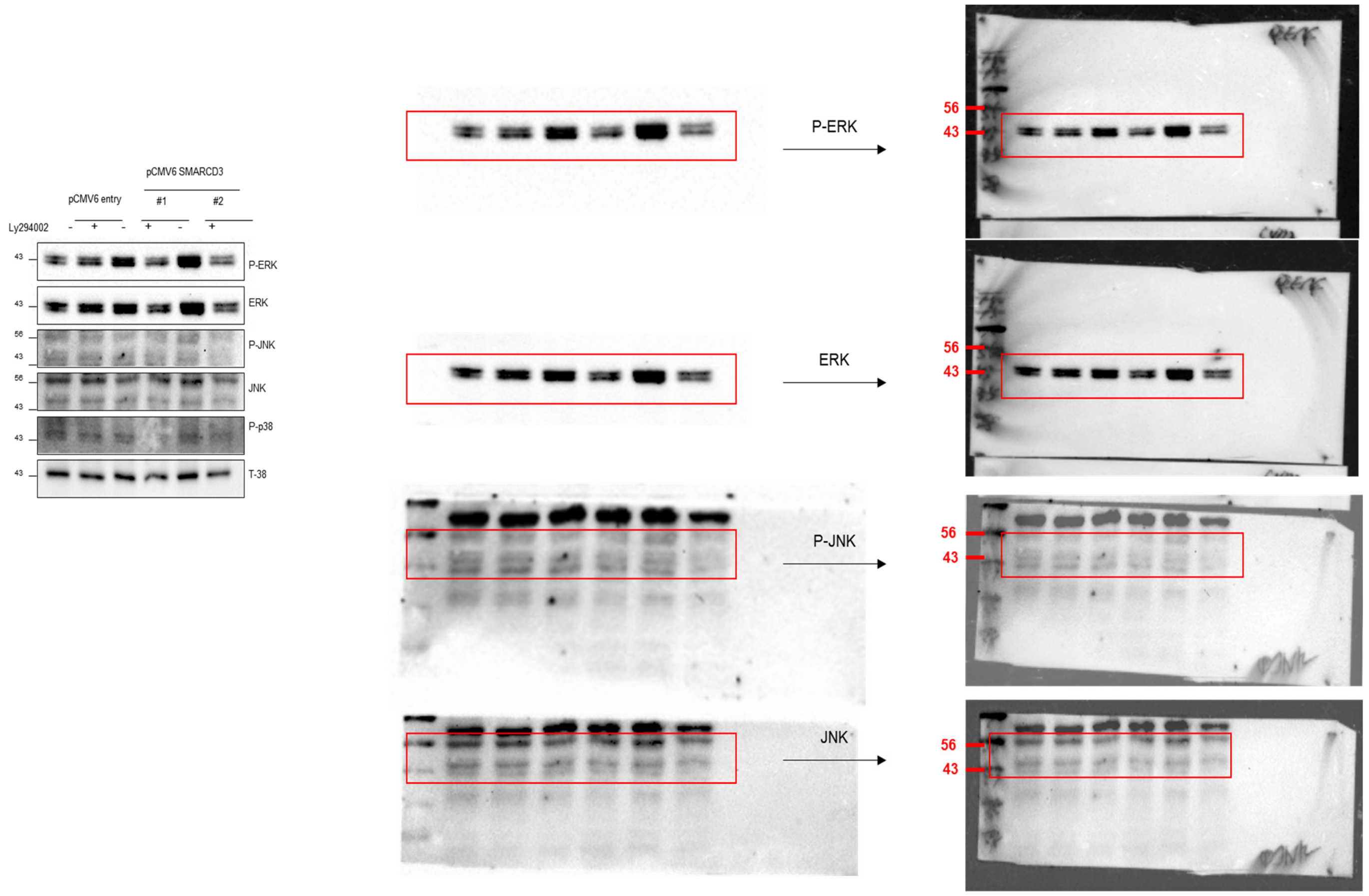
pCMV6 SMARCD3
pCMV6 entry
#1
#2
Ly294002
- + - + - +
43
56
P-ERK
ERK
P-JNK
JNK
P-p38
T-38
P-ERK
ERK
P-JNK
JNK
56
43

D

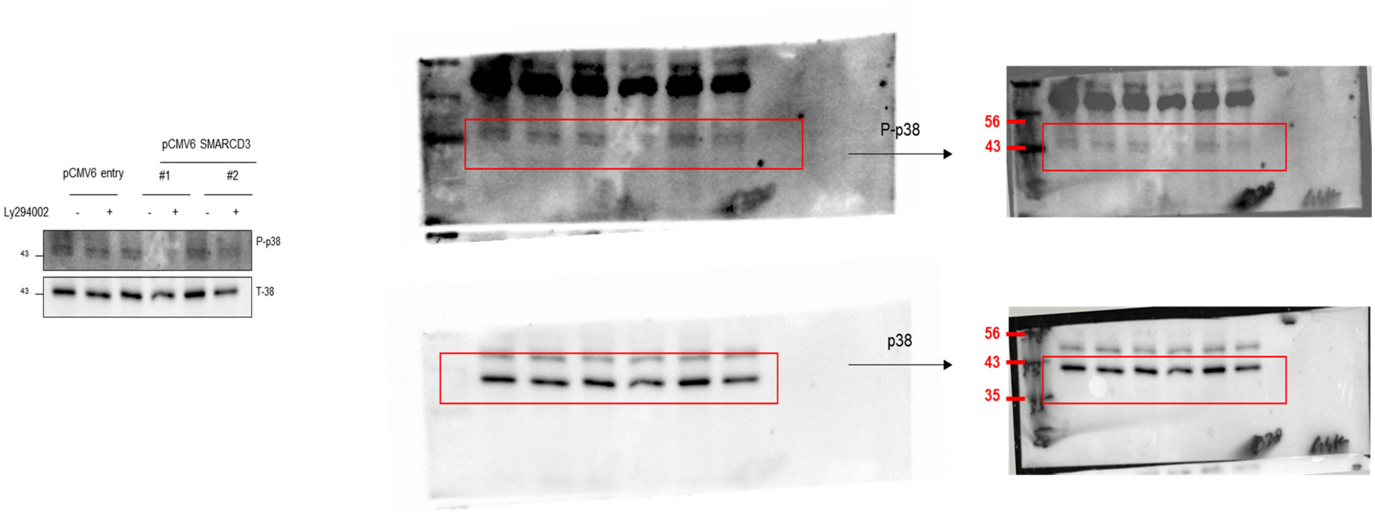

Figure S6. Original Western Blot Figures (A-D) of Figure 2E.

A

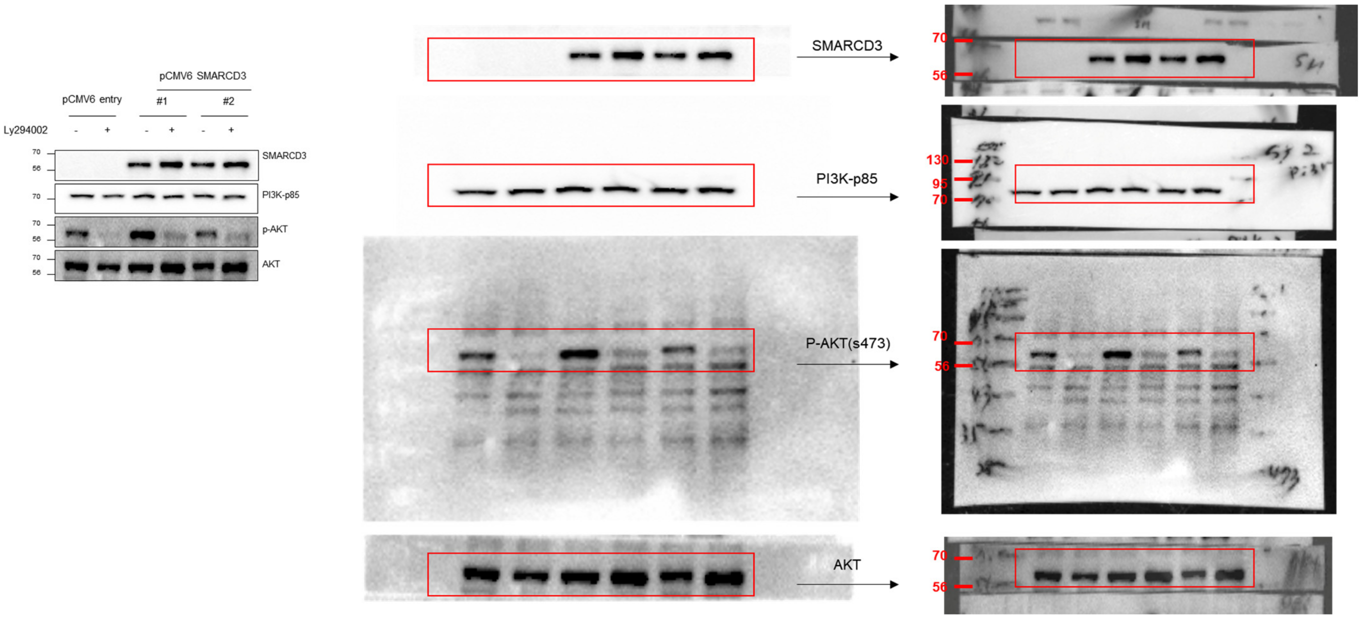

B

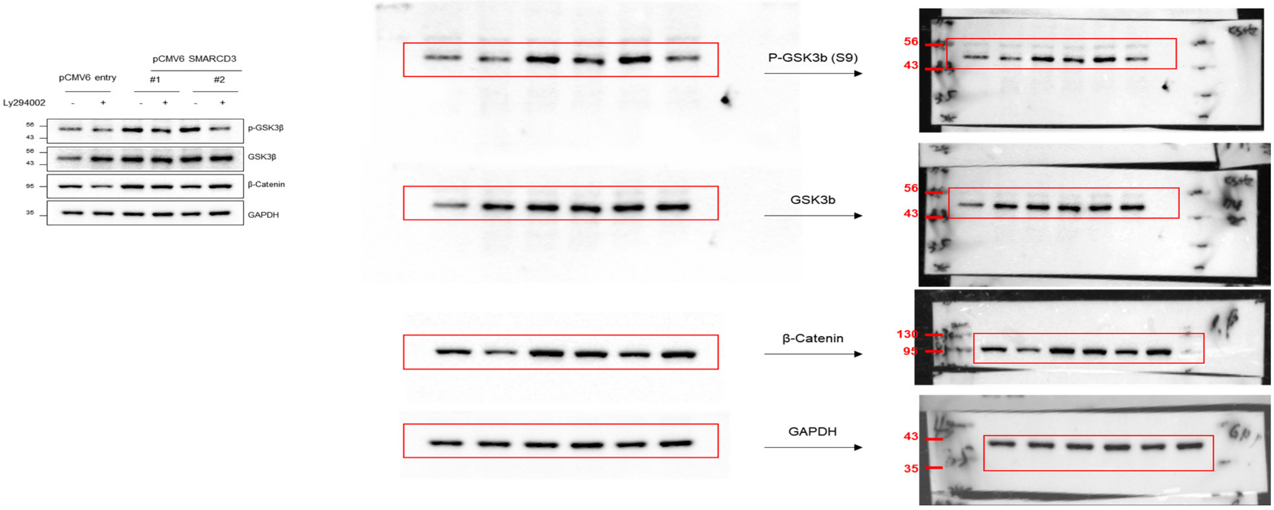

C

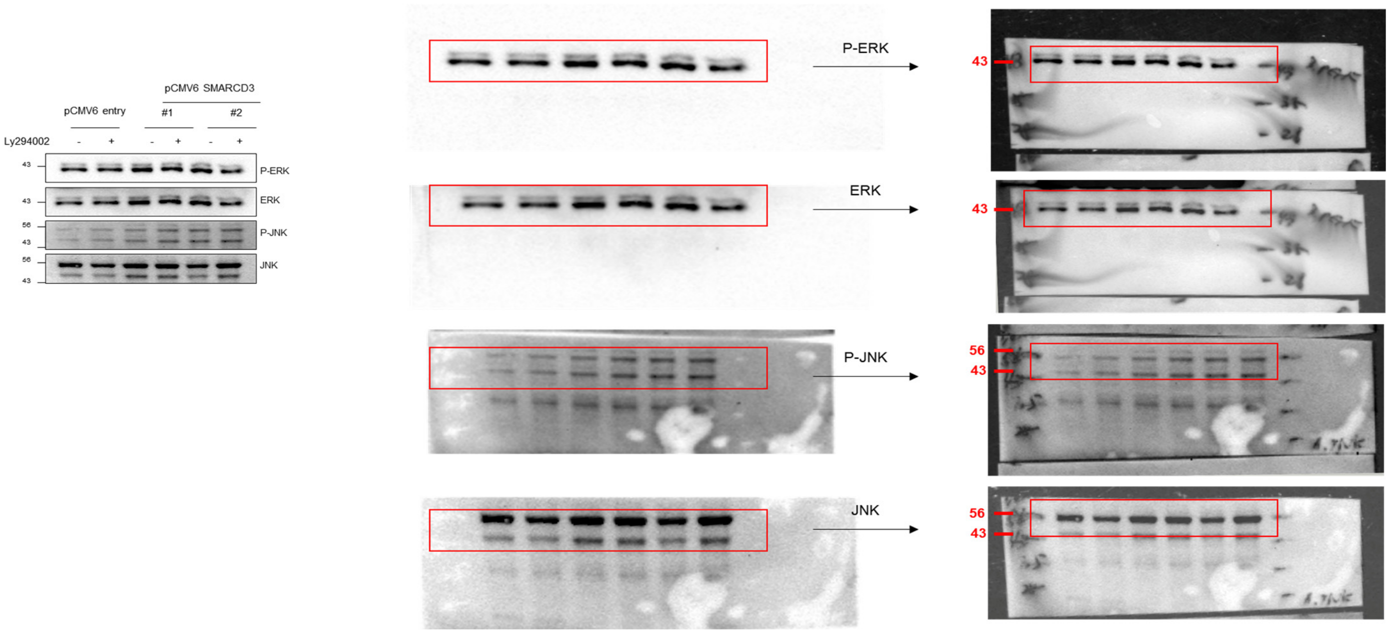

D

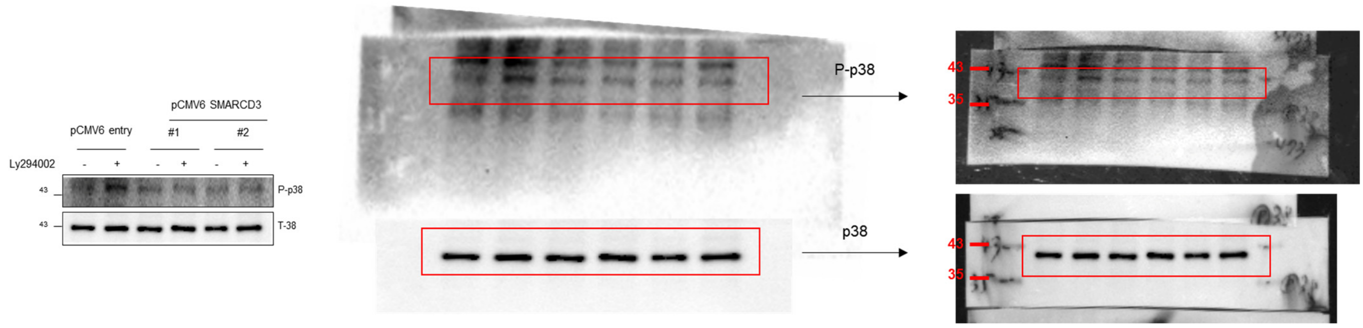

Figure S7. Original Western Blot Figures (A-D) of Figure 2F.

A

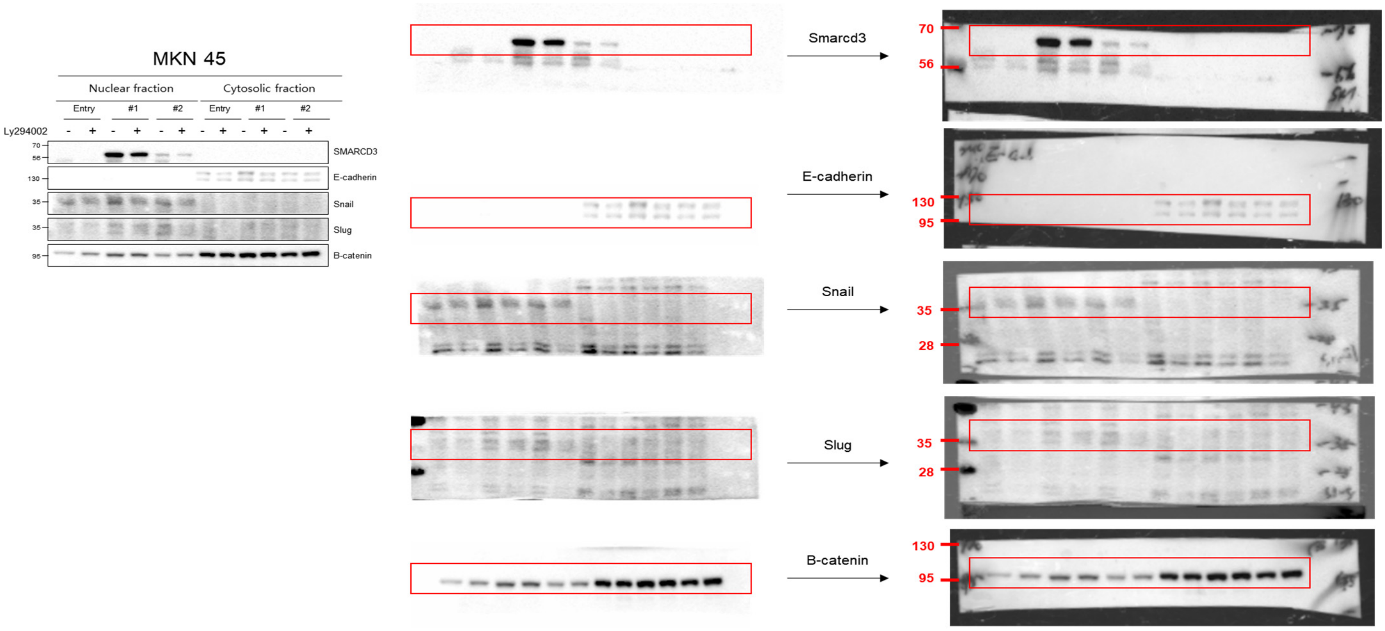

B

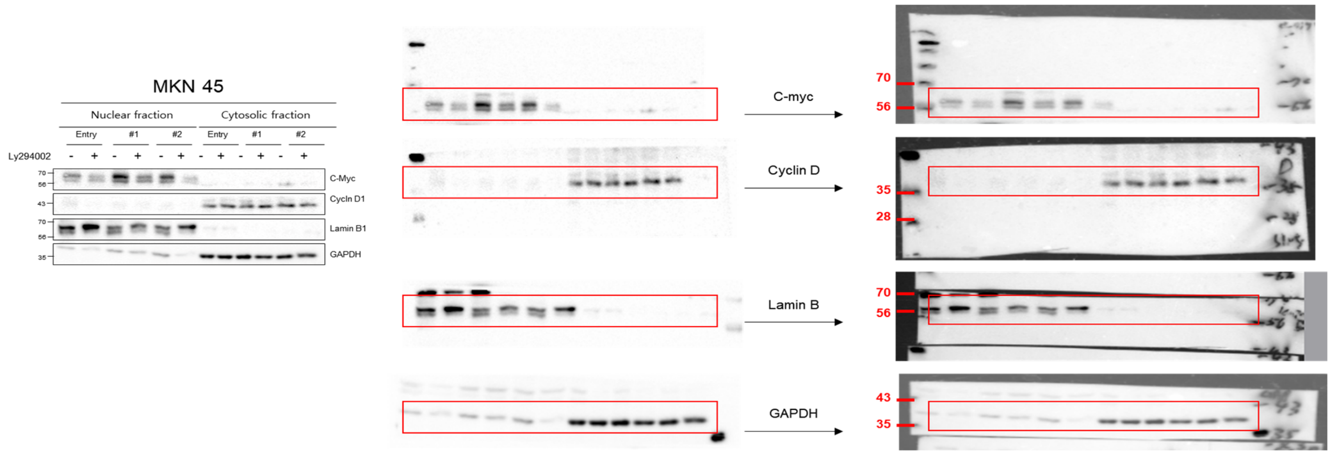

Figure S8. Original Western Blot Figures (A, B) of Figure 3A.

A

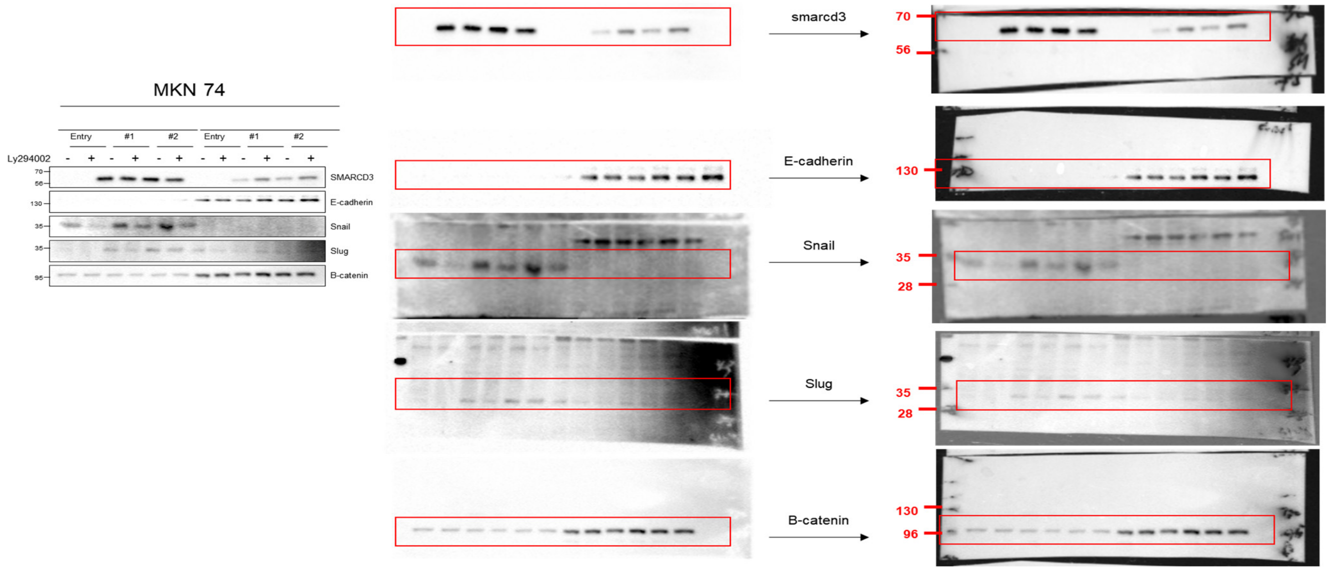

B

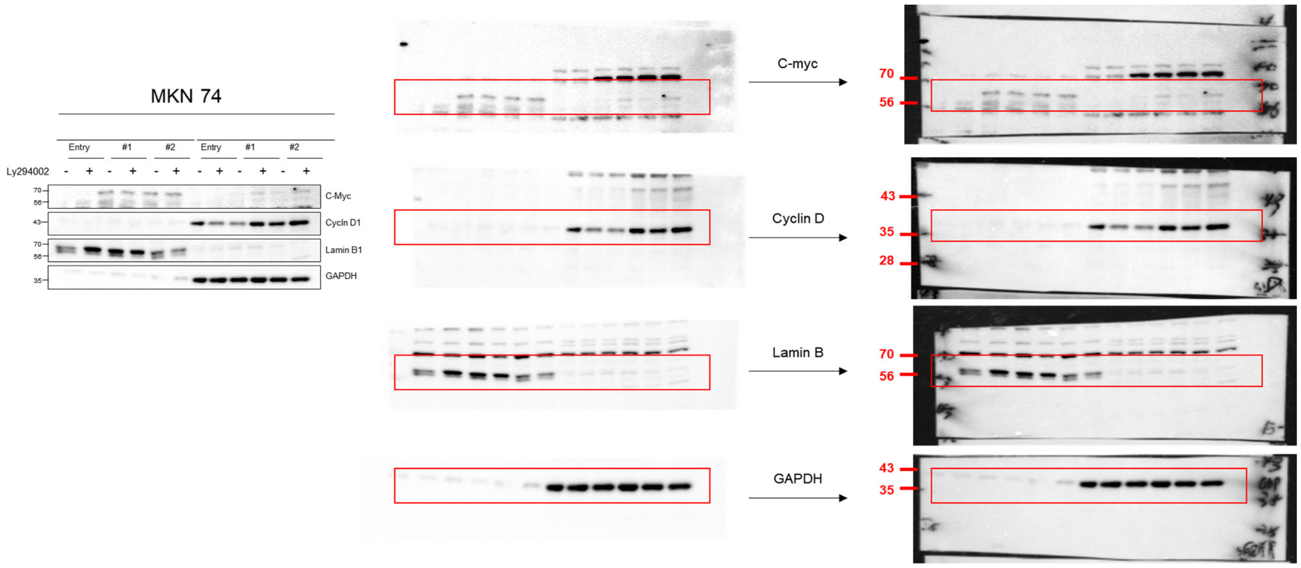

Figure S9. Original Western Blot Figures (A, B) of Figure 3B.

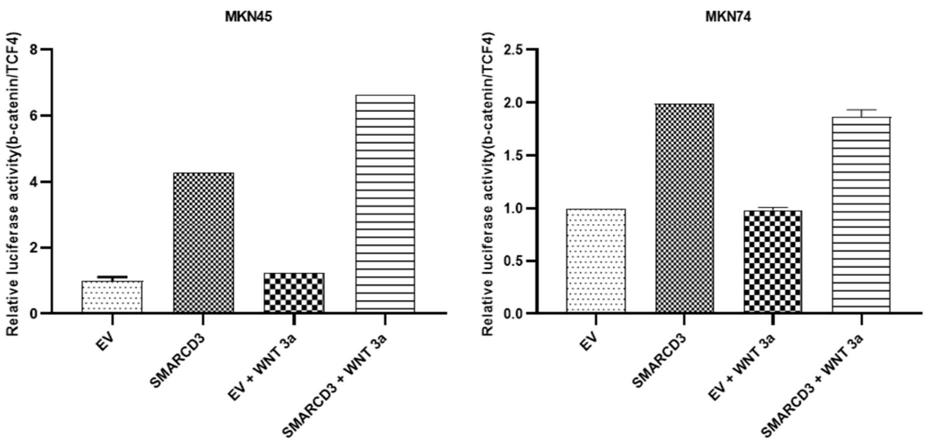

Figure S10. TOPFlash dual luciferase reporter assay. MKN45 and MKN74 cells were seeded at a density of 1×10^5 cells per well in 24-well plates and cultured overnight. Transfection was performed using Lipofectamine 2000 (Invitrogen) according to the manufacturer's instructions. Each well was co-transfected with 500 ng of M50 Super 8×TOPFlash plasmid (Addgene, Watertown, MA) and 100 ng of pRL-TK plasmid (Promega) to assess Wnt/β-catenin signalling activity using the Dual-Luciferase Reporter Assay System (Promega). TCF/LEF reporter analysis was performed 4 hours after treatment with recombinant human WNT3A (#5036-WN; R&D Systems, MN, USA). EV: control group, SMARCD3: smarcd3 overexpression, EV + WNT3A: WNT3 treatment in control cells, smarcd3 + WNT3A: WNT3A treatment in smarcd3-overexpressing cells.

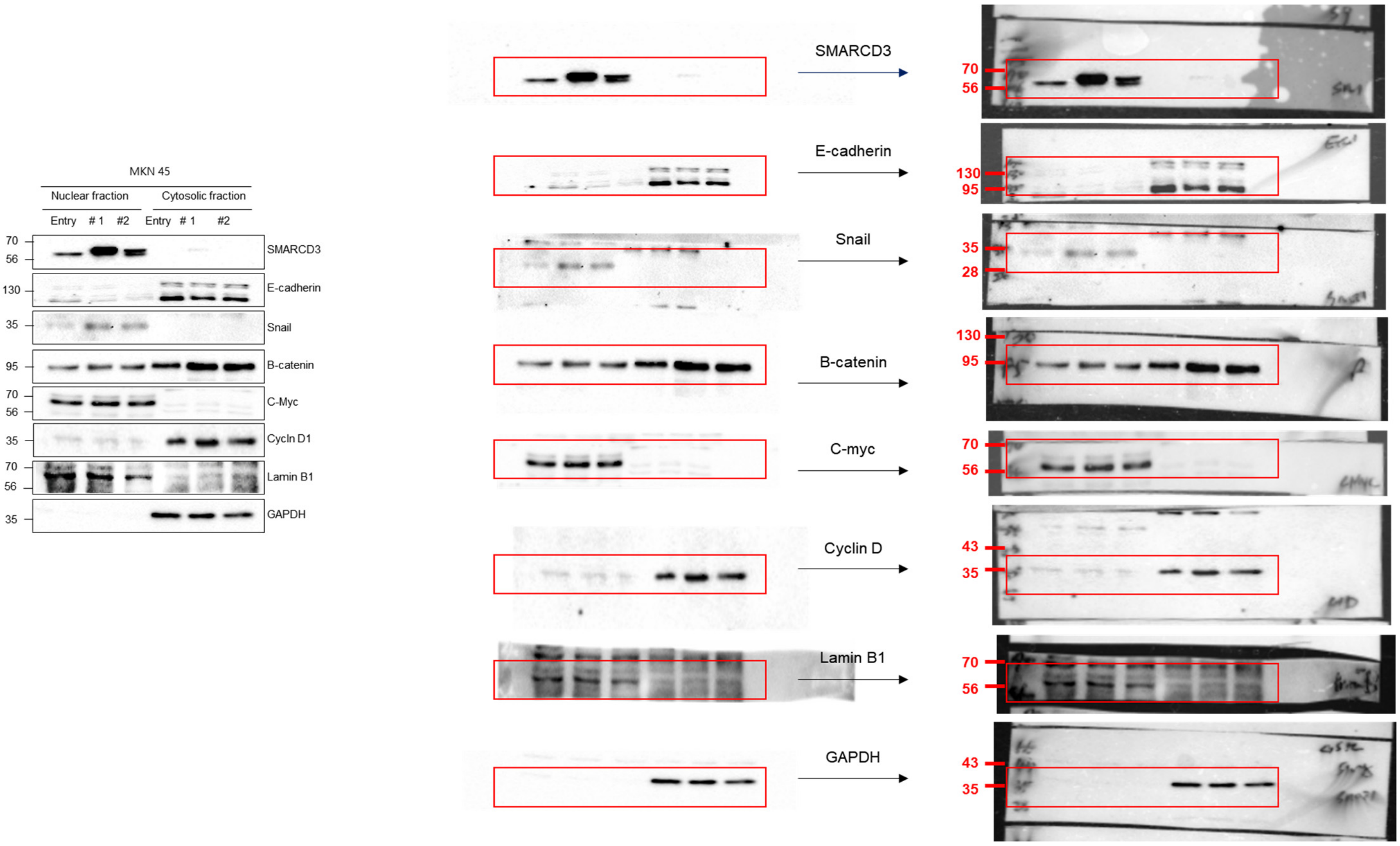

Figure S11. Original Western Blot Figures of Figure 5A.

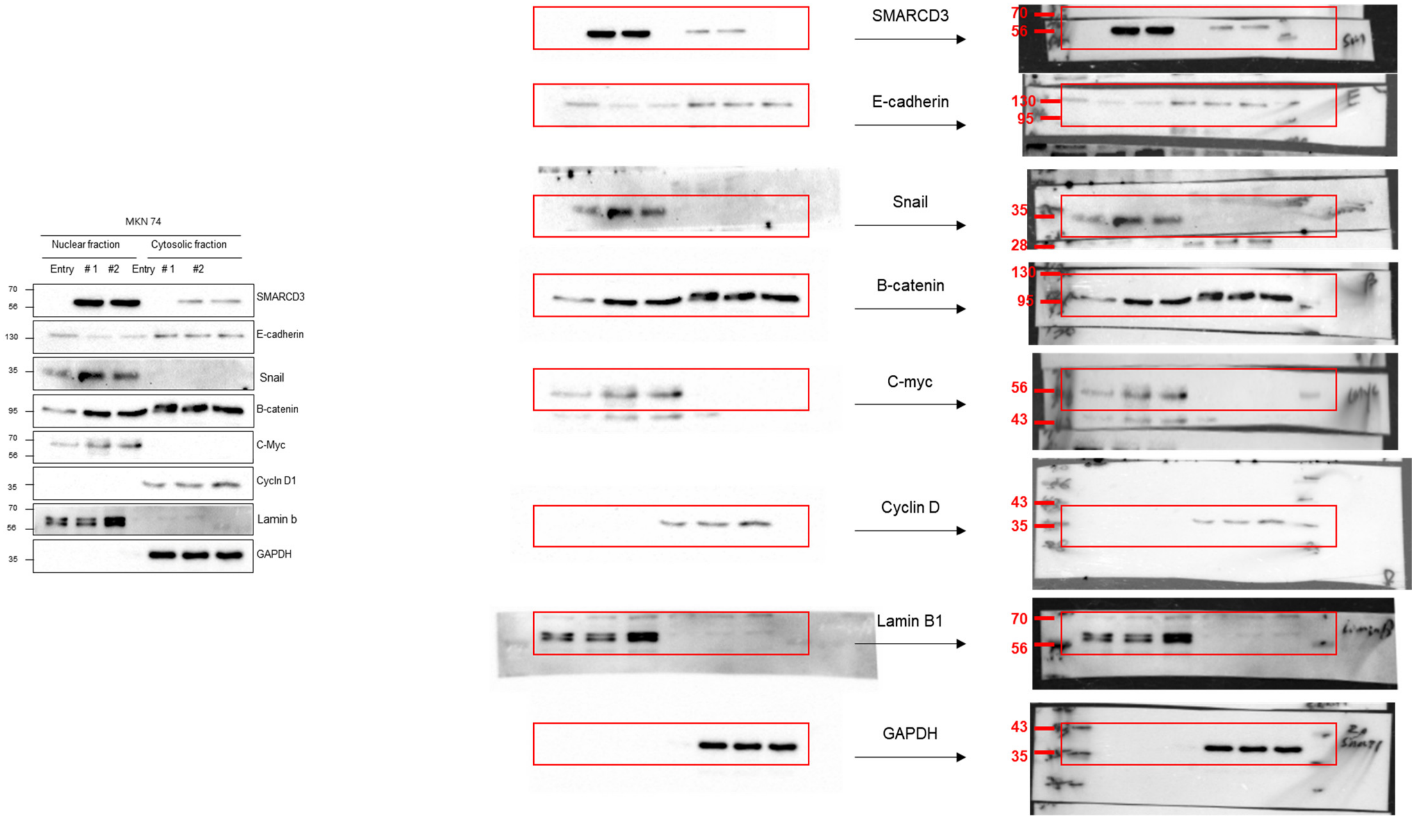

Figure S12. Original Western Blot Figures of Figure 5B.
